# Persistent Depressive Symptoms are Independent Predictors of Low-Grade Inflammation Onset Among Healthy Individuals

**DOI:** 10.5935/abc.20170080

**Published:** 2017-08

**Authors:** Fábio Gazelato de Mello Franco, Antonio Gabriele Laurinavicius, Paulo A. Lotufo, Raquel D. Conceição, Fernando Morita, Marcelo Katz, Maurício Wajngarten, José Antonio Maluf Carvalho, Hayden B. Bosworth, Raul Dias Santos

**Affiliations:** 1 Hospital Israelita Albert Einstein, São Paulo, SP - Brazil; 2 Centro de Pesquisa Clínica e Epidemiológica da Universidade de São Paulo (USP), São Paulo, SP - Brazil; 3 Instituto do Coração (InCor) - HCFMUSP, São Paulo, SP - Brazil; 4 Duke University Medical Center - USA

**Keywords:** Depression, Cardiovascular Diseases, Inflammation, Patient Selection

## Abstract

**Background:**

Depressive symptoms are independently associated with an increased risk of cardiovascular disease (CVD) among individuals with non-diagnosed CVD. The mechanisms underlying this association, however, remain unclear. Inflammation has been indicated as a possible mechanistic link between depression and CVD.

**Objectives:**

This study evaluated the association between persistent depressive symptoms and the onset of low-grade inflammation.

**Methods:**

From a database of 1,508 young (mean age: 41 years) individuals with no CVD diagnosis who underwent at least two routine health evaluations, 134 had persistent depressive symptoms (Beck Depression Inventory - BDI ≥ 10, BDI+) and 1,374 had negative symptoms at both time points (BDI-). All participants had been submitted to repeated clinical and laboratory evaluations at a regular follow-up with an average of 26 months from baseline. Low-grade inflammation was defined as plasma high-sensitivity C-Reactive Protein (CRP) concentrations > 3 mg/L. The outcome was the incidence of low-grade inflammation evaluated by the time of the second clinical evaluation.

**Results:**

The incidence of low-grade inflammation was more frequently observed in the BDI+ group compared to the BDI- group (20.9% vs. 11.4%; p = 0.001). After adjusting for sex, age, waist circumference, body mass index, levels of physical activity, smoking, and prevalence of metabolic syndrome, persistent depressive symptoms remained an independent predictor of low-grade inflammation onset (OR = 1.76; 95% CI: 1.03-3.02; p = 0.04).

**Conclusions:**

Persistent depressive symptoms were independently associated with low-grade inflammation onset among healthy individuals.

## Introduction

Depression is a prevalent disease that leads to considerable global burden and disabilities.^1^ The relationship between depressive symptoms and cardiovascular disease (CVD) has been well documented as it almost doubles the risk of developing coronary heart disease.^2,3^ Although traditional cardiovascular risk factors tend to cluster in depressed patients as a consequence of an unhealthy lifestyle (e.g., poor diet, lack of exercise), these unhealthy behaviors do not adequately account for the impact of depression on CVD.

Inflammation may act as a possible mechanistic link between depressive symptoms and CVD. Elevation in plasma high-sensitivity C-reactive protein (CRP) is a marker of a low-grade inflammatory state that has been associated with the incidence of CVD^4^ and all-cause mortality.^5^ Studies have reported an association between depressive symptoms and plasma CRP elevation in cross-sectional analyses.^6-8^

The aim of this study was to assess the association of persistent depressive symptoms with a low-grade inflammatory process, taking into account potential explanatory factors such as physical activity, obesity and sex, in a group of young, healthy individuals. As persistent depression symptoms would be independent predictors of low-grade inflammation among healthy and young individuals, this condition should be included in routine health evaluations to avoid future cardiovascular events.

## Methods

### Participants

From a dataset of 34,581 subjects, 4,222 individuals with at least two consecutive yearly exams were selected. All individuals had no background of CVD according to self-report. Of those, 1,508 individuals who did not have signs of low-grade inflammation, defined as CRP values < 3 mg/L at baseline (time point 1), were included. Depressive symptom presence was defined as Beck Depression Inventory (BDI) ≥ 10 scale points assessed at times 1 and 2. Subjects were divided into those who had (BDI+, n = 134, 8.8%) or not (BDI-, n = 1,374, 91.2%) persistent depressive symptoms. Exclusion criteria were the presence of chronic or acute inflammatory (defined as CRP > 10 mg/L at either time points) or previous cardiovascular diseases (defined as myocardial infarction, angina, coronary revascularization, stroke, peripheral artery disease or heart failure) according to self-declaration of health conditions.

### Independent Variables

As previously described, subjects were submitted to a routine mandatory health evaluation paid by their employers.^9^ All conditions included in the evaluations potentially associated with future low-grade inflammation, such as clinical background, smoking, physical activity, laboratory analyses (cholesterol, triglycerides, glucose, uric acid, creatinine, liver transaminases) and the presence of hepatic steatosis were considered as independent variables. Demographics, medical history and medication use were routinely recorded. Smoking status was categorized as current smoker (at least 1 cigarette during the last 30 days) versus current nonsmoker. The International Physical Activity Questionnaire (IPAQ)^10^ was used to assess physical activity level. Blood pressure was measured 3 times at the sitting position with an aneroid sphygmomanometer according to the standard method recommended by the American Heart Association.^11^ Hypertension was defined according to current guidelines.^12,13^ Height (meter) and weight (kilogram) were measured with a standard physician's scale and a stadiometer to calculate body mass index (BMI, kg/m^2^). Waist circumference was recorded at the smallest diameter between the iliac crest and the costal margin with a plastic anthropometric tape held parallel to the ground.

Blood samples were collected after at least 12 hours fasting and processed at the Central Laboratory of the Preventive Medicine Unit of the Hospital Israelita Albert Einstein, Sao Paulo, Brazil. Total cholesterol, triglycerides (TG), HDL-cholesterol, glucose, uric acid, creatinine, and liver transaminases were determined using standardized automated laboratory tests (Vitros 5600, Johnson & Johnson Orthoclinical Diagnostics). When TG < 400 mg/dL, LDL-cholesterol was calculated by the Friedwald formula. When TG ≥ 400 mg/dL, LDL-cholesterol levels were measured directly. High-sensitivity CRP concentrations were determined by immunonephelometry (Dade-Behring). Hepatic steatosis was identified by the presence of an ultrasound pattern of bright liver, with evident contrast between hepatic and renal parenchyma as previously described.^14^ Excess body weight was defined by the presence of a BMI > 25 kg/m^2^, while abdominal obesity was characterized by elevated waist circumference (> 88 cm in women and > 102 cm in men). Metabolic syndrome was defined by the joint AHA/IDF consensus.^15^

### Depression symptoms assessment

The BDI was used for depression symptom assessment^16^ and repeated each time individuals underwent a new check-up survey. In brief, BDI is a 21-item self-administered scale with four alternative statements for each item, scoring from 0 to 3 points and a maximum score of 63 points. Similarly to other studies, scores ≥10 points were suggestive of depression (BDI+), with higher points indicative of increasing depression severity.^17,18^ Those with scores < 10 were not considered with depressive symptoms (BDI-).

### Outcome

The outcome was the incidence of new cases of inflammation according to onset of elevated CRP concentrations.

### IRB approval

The Institutional Human Research Committee approved this study and all participants provided written informed consent to allow for the use of their medical information, as outlined by the 1975 Helsinki Declaration.

### Statistical analysis

Continuous data with normal distribution are expressed as mean and standard deviation. Continuous data with non-normal distribution are represented as median and interquartile range. For normality hypothesis, the Kolmogorov-Smirnov test was adopted. Categorical data are expressed by percentage and comparison was made by the chi- square test. For normally distributed continuous data, Student *t* test was adopted. Non-normally data are analyzed by Mann-Whitney test. A BDI score ≥ 10 points was adopted to identify individuals with significant depressive symptoms.^18^ CRP was considered as a dichotomous variable and results > 3 mg/L were considered positive for low-grade inflammation and cardiovascular risk.^19^ The chi-square model was used to analyze the association between depressive symptoms and CRP. Logistic regression was used to determine the effect of depressive symptoms on low-grade inflammation after adjusting for potential confounding variables, such as age, sex, BMI, blood pressure, total cholesterol, smoking, diabetes, hepatic steatosis, physical activity and metabolic syndrome. Statistical significance was inferred at a two-tailed p < 0.05. All analyses were performed using SPSS v 20.0 (SPSS, Inc, Armonk, NY, USA).

## Results

This was a predominantly young, Caucasian, male population with low calculated risk of CVD. The mean (standard deviation) follow-up time was 26 ± 10 months. Table 1 shows the clinical and laboratory characteristics of individuals presenting with and without depressive symptoms (BDI+ and BDI-) at time point 1. There was a greater prevalence of females (30.6% vs 18.4%; p = 0.001) and physical inactivity (25.5% vs 16.2%; p = 0.015) in the BDI+ group relative to the non-depressed group. Also, those with depressive symptoms had higher plasma TG (p = 0.008) and lower plasma levels of creatinine (p = 0.009) relative to those with no depressive symptoms. Of importance, no difference in age, BMI, waist circumference, smoking, metabolic syndrome prevalence and CRP levels was observed between the groups.

**Table 1:** Clinical and laboratory characteristics of subjects presenting (BDI+) or not (BDI-) persistent depressive symptoms at baseline

	No depressive symptoms (BDI-) n = 1,374	Depressive symptoms (BDI+) n = 134	p value
Age, mean (SD)	41.4 (8)	40.4 (6.5)	0.081*
Sex (%female)	18.4%	30.6%	0.001¥
Smoking (%)	6.3%	8.9%	0.245¥
Hypertension (%)	9.5%	7.5%	0.432¥
Diabetes (%)	2.3%	2,2%	0.999¥
BMI (kg/m^2^)	26.1 (3.6)	26.6 (3.7)	0.114*
Waist circumference, mean (SD)	92.3 (11.1)	92.4 (12.2)	0.921*
Metabolic Syndrome (%)	14.7	20	0.111¥
Physical inactivity (%)	16.2	25.5	0.015¥
Lipid-lowering agents (%)	11.9	10.4	0.627¥
Oral antidiabetic drug or insulin use (%)	3.4	6.7	0.087¥
Glucose, mean (SD)	88 (12.8)	89 (14)	0.544*
LDL-C, mean (SD)	126 (34.4)	127 (39.8)	0.749*
HDL-C, mean (SD)	49 (12.7)	49 (12.5)	0.607*
Total cholesterol, mean (SD)	201 (37.3)	206 (42.5)	0.177*
Triglycerides, median (IQR)	108 (79; 154)	129 (92; 208)	0.003£
Creatinine, mean (SD)	0.87 (0.19)	0.82 (0.23)	0.009*
CRP, median (IQR)	1.00 (0.5;1.8)	1.15 (0.5; 2.3)	0.041£
Steatosis (%)	34.4	44.6	0.020¥

Data were expressed as mean (SD) or median (IQR) for normal and non-normal data respectively. IQR: interquartile ranges; SD: standard deviation; age- in years; body mass index- BMI in kg/m^2^; waist circumference- in cm; plasma lipids, glucose and creatinine- in mg/dL; C reactive protein- in mg/L;

* Student t test;

¥ Chi-square test,

£ Mann-Whitney test.

Table 2 shows the clinical and laboratory characteristics of subjects presenting with and without low-grade inflammation at time point 2. Low-grade inflammation was detected respectively in 20.9% and 11.4% of participants in the BDI+ and BDI- groups (OR = 2.05; 95% CI: 1.31-3.21; p < 0.001). In bivariate analysis, physical activity (p = 0.049), depressive symptoms (p < 0.001), metabolic syndrome (p = 0.017), waist circumference (p < 0.001), and BMI (p < 0.001) were also associated with low-grade inflammation.

**Table 2:** Bivariate clinical and laboratory characteristics associated with the presence or not of low-grade inflammation (CRP > 3 mg/L) at exam time point 2

Parameter	CRP ≤ 3	CRP > 3 mg/L	p value
Age, mean (SD)	41.4 (7.9)	40.8 (7.6)	0.395*
**Sex, n (%)**			
Female	248 (84.7)	45 (15.3)	0.077¥
Male	1074 (88.5)	140 (11.5)	
**Smoking, n (%)**			
No	1234 (87.8)	172 (12.2)	0.793¥
Yes	86 (86.9)	13 (13.1)	
**Physical activity, n (%)**			
Yes	876 (88.8)	111 (11.2)	0.049¥
No	171 (83.8)	33 (16.2)	
**Depressive symptoms, n (%)**			
BDI+	1217 (88.6)	157 (11.4)	0.001¥
BDI-	106 (79.1)	28 (20.9)	
**Metabolic syndrome, n (%)**			
No	1097 (88.6)	141 (11.4)	0.017¥
Yes	184 (82.9)	38 (17.1)	
**Hypertension, n (%)**			
No	1197 (87.6)	170 (12.4)	0.536¥
Yes	126 (89.4)	15 (10.6)	
**Diabetes, n (%)**			
No	1293 (87.7)	181 (12.3)	0.999¥
Yes	30 (88.2)	4 (11.8)	
**Hepatic steatosis, n (%)**			
No	839 (88.7)	107 (11.3)	0.115¥
Yes	443 (85.9)	73 (14.1)	
**Lipid-lowering drugs, n (%)**			
No	1166 (87.6)	165 (12.4)	0.676¥
Yes	157 (88.7)	20 (11.3)	
**Antidiabetic drugs or insulin, n (%)**			
No	1277 (87.9)	175 (12.1)	0.194¥
Yes	46 (82.1)	10 (17.9)	
BMI, mean (SD)	25.9 (3.5)	27.4 (3.9)	< 0.001*
Waist circumference, mean (SD)	91.9 (11.1)	95.2 (11.2)	< 0.001*
Total cholesterol, mean (SD)	201.3 (37.6)	204.4 (39.1)	0.291*
LDL-C, mean (SD)	126 (34.6)	128.5 (36.6)	0.372*
HDL-C, mean (SD)	49.3 (12.7)	48.4 (12)	0.368*
Triglycerides, median (IQR)	108 (80;154)	118 (95;174)	0.019£
Glucose, mean (SD)	87.9 (12.8)	88.3 (13.4)	0.677*
Creatinine, mean (SD)	0.87 (0.19)	0.86 (0.18)	0.692*

Age: in years; body mass index: BMI in kg/m^2^; waist circumference- in cm; plasma lipids, glucose and creatinine- in mg/dL; C-reactive protein- in mg/L; IQR: interquartile range; SD: standard deviation;

* Student t test;

¥ Chi: square test,

£ Mann Whitney test.

Confounding factors and results obtained in the bivariate analysis were included in the multivariate analysis. In this analysis, the association of depressive symptoms and low-grade inflammation was adjusted for age, sex, waist circumference, BMI, levels of physical activity, smoking, presence of hepatic steatosis, and metabolic syndrome prevalence. New cases of inflammation were associated with depressive symptoms regardless those variables mentioned above (OR = 1.76; 95% CI: 1.03-3.02; p = 0.04). The statistical power to infer a difference on BDI+ group compared to BDI- was 56.5%, with a two-sided level of significance of 0.05.

## Discussion

A positive association between persistence of depressive symptoms and low-grade inflammation after a 2-year average follow-up was observed. Findings were robust even after adjusting for risk factors associated with elevation in plasma CRP levels, such as abdominal obesity and metabolic syndrome.

Atherosclerosis, the main pathological substrate of CVD, is a chronic degenerative disorder with a low-grade inflammatory component. Persistent depressive symptoms over at least 2 years have been prospectively associated with coronary artery calcification detected by computed tomography,^20^ a surrogate marker of atherosclerosis burden and a robust marker of cardiovascular event risk.^21^ Robust evidence from prospective studies shows a clear and independent association of elevated CRP levels with cardiovascular events and mortality.^4,5^ Indeed, increased CRP levels have been shown to add modest, but significant ability to improve risk reclassification over traditional risk markers in asymptomatic individuals.^22^ The results of this study suggest that depressive symptoms are associated not only with atherosclerotic plaque burden, as previously shown,^20,23^ but also with the low-grade inflammatory component of atherosclerosis. Therefore, detection of depressive symptoms might have prognostic information for CVD risk evaluation.

This is one of the largest longitudinal studies examining persistence depressive symptoms and subsequent inflammation onset in a young non-CVD population. The strength of the current study is the comprehensive clinical, laboratory and behavioral factors that may be associated with the depression-inflammation relationship. These factors include physical inactivity, obesity and smoking. Another strength of this study was the enrollment of a poorly studied population, composed by subjects without previous CVD. Despite this sample of non-CVD individuals, persistent depressive symptoms were associated with subsequent inflammation. This finding highlights the importance of depression on cardiovascular primary prevention in a young population.

As previously described, depressive symptoms were associated with clinical characteristics associated with elevated plasma CRP levels, such as female sex and increased adiposity.^9,22^ However, there is controversy if female sex may be associated with the process of low-grade inflammation as a result of depression.^24-26^ While one study has found depressed white women to be more susceptible to inflammation,^24^ other studies^25,26^ have found an association between depression and inflammation only in men. In contrast, in this study the association of depressive symptoms and elevated CRP levels persisted even after adjustment for sex.

Some unhealthy behaviors are associated with depression and may interfere with the inflammation-depression relationship. In a cohort of 667 outpatients with established coronary heart disease from the Heart and Soul Study, depressive symptoms predicted inflammation after 5 years of follow-up.^27^ However, this association was no longer significant after adjustment for physical inactivity, smoking and higher BMI, which suggests that other behavioral factors may be important modulators of the depression-inflammation process. The same inference was reached by the authors of a prospective study of 289 patients with atrial fibrillation,^28^ in which obesity was the single strongest predictor of inflammation, eliminating the association between depression and inflammation in multivariate analyses. In contrast, in a group of 3,609 men and women with a mean age of 60.5 from the English Longitudinal Study of Ageing, Hamer et al. have found that baseline depression was associated with further inflammation 2 years later, even after taking into account other behavioral factors.^29^ That study corroborates our own results, in which a persistent relationship between depressive symptoms and inflammation was observed after adjustment for age, sex, smoking status, physical inactivity, metabolic syndrome, fatty liver and excess body weight. Indeed, the adjustment for fatty liver is very important since we had previously shown a strong and independent association of hepatic steatosis, a highly active visceral fat depot, with elevated plasma CRP levels independently of markers of obesity in apparently healthy subjects.^9^ Another point of interest in the current study was that most individuals had mild depressive symptoms. However, even this burden of depression symptoms may lead to future low-grade inflammation.

### Limitations

The findings were limited to the inclusion of a predominantly Caucasian and young population. However, we did observe that the effects were robust across both sexes. Patients with depressive symptoms might have a lower adherence to subsequent exams, increasing the dropout of BDI+ subjects after the first time set. This lack of adherence during follow-up would have an impact on the statistical power of the study analysis. Finally, plasma CRP levels were measured only twice in this study, however in the absence of clear inflammatory diseases, high-sensitivity CRP assays have shown to have a good reproducibility and low variability.^30^

## Conclusions

In summary, our results demonstrate that persistent depressive symptoms are an independent predictor of future low-grade inflammation onset in this population. These findings suggest that depressive symptoms should be considered among important factors contributing to subsequent health problems and should therefore be screened, even in apparently healthy individuals undergoing routine healthcare exams.
