# Erratum

**DOI:** 10.5935/abc.20170117

**Published:** 2017-08

Arq Bras Cardiol. 2017; [online].ahead print, PP.0-0

Consider correct authors' affiliations Fábio Gazelato de Mello Franco,^1^ Antonio Gabriele Laurinavicius,^2^ Paulo A. Lotufo,^3^ Raquel D. Conceição,^2^ Fernando Morita,^1^ Marcelo Katz,^1^ Maurício Wajngarten,^1^ José Antonio Maluf Carvalho,^1^ Hayden B. Bosworth,^5^ Raul Dias Santos^2,4^ to the institutions: Hospital Israelita Albert Einstein;^1^ Centro de Medicina Preventiva e Programa de Cardiologia do Hospital Israelita Albert Einstein;^2^ Centro de Pesquisa Clínica e Epidemiológica da Universidade de São Paulo (USP);^3^ Unidade Clínica de Lípides Instituto do Coração (InCor) do Hospital das Clínicas da Faculdade de Medicina da USP;^4^ São Paulo, SP - Brasil; Duke University Medical Center^5^ - EUA to the article "Persistent Depressive Symptoms are Independent Predictors of Low-Grade Inflammation Onset Among Healthy Individuals", published ahead of print.

